# Surgical antibiotic prophylaxis: is the clinical practice based on evidence?

**DOI:** 10.31744/einstein_journal/2020AO5427

**Published:** 2020-11-12

**Authors:** Lucas Borges Pereira, Cinara Silva Feliciano, Diego Silva Siqueira, Fernando Bellissimo-Rodrigues, Leonardo Régis Leira Pereira

**Affiliations:** 1 Universidade de São Paulo Faculdade de Ciências Farmacêuticas de Ribeirão Preto Ribeirão PretoSP Brazil Faculdade de Ciências Farmacêuticas de Ribeirão Preto, Universidade de São Paulo, Ribeirão Preto, SP, Brazil.; 2 Universidade de São Paulo Hospital das Clínicas Faculdade de Medicina de Ribeirão Preto Ribeirão PretoSP Brazil Hospital das Clínicas, Faculdade de Medicina de Ribeirão Preto, Universidade de São Paulo, Ribeirão Preto, SP, Brazil.; 3 Universidade Estadual Paulista “Júlio de Mesquita Filho” Faculdade de Ciências Farmacêuticas AraraquaraSP Brazil Faculdade de Ciências Farmacêuticas, Universidade Estadual Paulista “Júlio de Mesquita Filho”, Araraquara, SP, Brazil.; 4 Universidade de São Paulo Faculdade de Medicina de Ribeirão Preto Ribeirão PretoSP Brazil Faculdade de Medicina de Ribeirão Preto, Universidade de São Paulo, Ribeirão Preto, SP, Brazil.

**Keywords:** Drug utilization review, Antibiotic prophylaxis, Drug resistance, microbial, Surgical wound infection/prevention & control, Infection control

## Abstract

**Objective:**

To assess the surgical antibiotic prophylaxis.

**Methods:**

This was a descriptive study performed at a public tertiary care university hospital gathering prescription, sociodemographic and hospitalization data of inpatients admitted in 2014 who used antimicrobial drugs. This data were obtained from the hospital electronic database. The antimicrobial data were classified according to the anatomical, therapeutic chemical/defined daily dose per 1,000 inpatients. An exploratory analysis was performed using principal component analysis.

**Results:**

A total of 5,182 inpatients were prescribed surgical antibiotic prophylaxis. Of the total antimicrobial use, 11.7% were for surgical antibiotic prophylaxis. The orthopedic, thoracic and cardiovascular postoperative units, and postoperative intensive care unit comprised more than half of the total surgical antibiotic prophylaxis use (56.3%). The duration of antimicrobial use of these units were 2.2, 2.0, and 1.4 days, respectively. Third-generation cephalosporins and fluoroquinolones had the longest use among antimicrobial classes.

**Conclusion:**

Surgical antibiotic prophylaxis was inadequate in the orthopedic, postoperative intensive care, thoracic and cardiovascular postoperative, gynecology and obstetrics, and otolaryngology units. Therefore, the development and implementation of additional strategies to promote surgical antibiotic stewardship at hospitals are essential.

## INTRODUCTION

Although surgical site infection (SSI) is easily avoided if the prevention criteria are rigorously followed, it remains a frequent healthcare-associated infection that is expensive for the public health system.^(^^1^^)^

One method to avoid SSI is surgical antibiotic prophylaxis (SAP), which has been shown to be effective in the scientific literature.^(^^2^^,^^3^^)^ However, the benefit is obtained only when the following criteria are used correctly: pre-surgical administration; adequate antimicrobial selection based on the surgical procedure and the pathogen most frequently observed in SSI; administration of more than one dose in procedures of longer duration; and discontinuing the dose after surgical wound closure.^(^^1^^,^^4^^)^

Nevertheless, SAP has been prescribed for long durations, or extended-spectrum antimicrobial are prescribed, which provide no additional benefit for specific surgeries or surgeries without indication. Zhang et al.,^(^^5^^)^ observed that SAP was administered 2 hours before surgical incision, with a utilisation duration ranging from 1 to 14 days. Queiroz et al.,^(^^6^^)^ reported that only 3.3% of SAP prescriptions were without error. Even with hospital SAP protocols, there are discrepancies in antimicrobial utilisation, as reported by Khakhkhar et al.,^(^^7^^)^ and Schmitt et al.^(^^8^^)^

The efficacy of SAP is determined by plasma concentrations of antimicrobial agents that prevent microbial growth during surgical procedures. Prolonged use after surgery does not provide additional benefit in SSI prevention and is associated with antimicrobial resistance.^(^^1^^,^^9^^)^

Antimicrobial resistance has been discussed by public health and government agencies. The 71^st^ General Assembly of the United Nations discussed the spread of antimicrobial-resistant infections and the World Health Organization (WHO) released the Antimicrobial Resistance: Global Report on Surveillance, highlighting the importance of the rational utilisation of antimicrobials to avoid resistance.^(^^1^^,^^10^^)^

The few reports on SAP utilisation, in addition to this context of antimicrobial resistance, underscores the need for studies on drug utilisation as a strategy to their rational, aiming to improve effectiveness of surgical prophylaxis and decrease the selection for antimicrobial resistance.

## OBJECTIVE

To describe the surgical antibiotic prophylaxis utilisation and to evaluate if it is in accordance with international guidelines.

## METHODS

### Subjects and setting

This descriptive study was performed at a 706-bed tertiary care teaching hospital in the state of São Paulo, Brazil. In this setting, patient information is managed by electronic systems, including the Electronic Prescription and System of Support to Hospital Care. The studied population comprised all inpatients aged ≥18 years who received antimicrobial prescription between January 1^st^ and December 31^st^, 2014.

### Data collection

The hospital electronic systems were reviewed and data on all antimicrobials prescribed in 2014 were collected. In this study, only antimicrobials prescribed for prophylaxis were analyzed, used to prevent infections in inpatients, such as SSI in surgical inpatients (this information was presented in electronic system data). The collected prescription information included the dose, time of utilization, specialty ward in which the drug was prescribed, and patient name and hospital identification. The clinical data included length of stay, clinical diagnosis according to International Statistical Classification of Diseases and Related Health Problems (ICD-10), and the number of inpatients-day, in 2014, for each specialty ward.

### Statistical analyses

A descriptive analysis of the inpatient data was performed. The results of descriptive analyses were presented as absolute and relative frequencies, mean, and standard deviation (SD).

Antimicrobial utilisation was calculated as the defined daily dose (DDD) per 1,000 inpatients-day. This calculation was performed for each drug used in each specific specialty ward.^(^^11^^)^

$$\text{DDD}/1,000\text{inpatients day}=\frac{\text{Sum of antimicrobial used in 2014}\left(\text{in grams}\right)}{\text{DDD of antimicrobial}\times \text{inpatients}-\text{day from specialty ward in 2014}}\times 1,000$$

The antimicrobials were classified according to anatomical therapeutic chemical (ATC) classification standardized by WHO.^(^^12^^)^

To complement the antimicrobial utilisation analysis, an exploratory analysis of data was performed by principal component analysis (PCA) using Statistica^®^ 7.0. The variables included in this analysis were the duration of drug utilisation, DDD/1,000 inpatients day, patient age, and length of stay. Principal component analysis is an exploratory multivariate statistical technique. It uses a matrix analysis process to convert a dataset with many variables into a new dataset represented by vectors. These new vectors represent the interaction between the different variables, reducing the amount of data to be analyzed, without losing the representativeness of the original database. After standardisation, the data were coded as zero for the mean value, and one for variance. The goal is to reduce the dimensionality of the data so that it is represented geometrically.

The study database has variables and antimicrobial consumption in the wards for each ATC class. If we were to represent this antimicrobial consumption of each ward on a graph, we would need a multidimensional graph (multiple axes), since each ATC class is a dimension.

**Figure 1:**
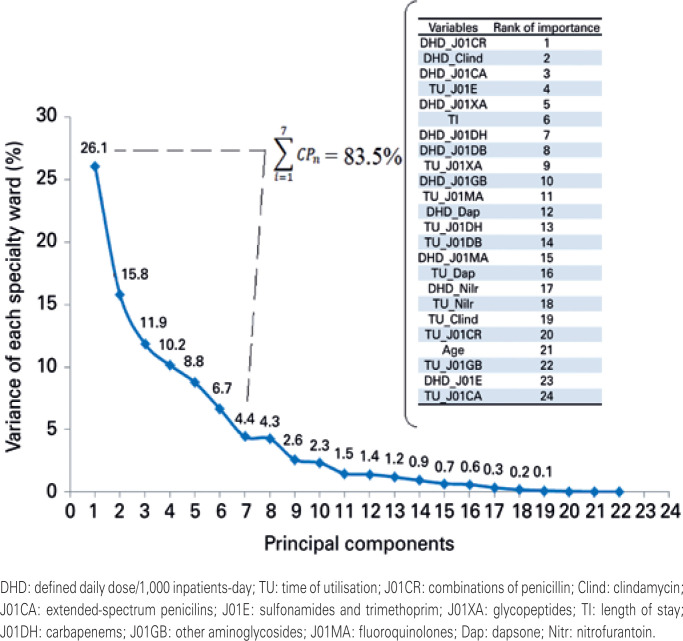
Variance of specialty wards (A) explained by the principal components, represented by the consumption of each antibiotic group (B)

**Figure 2:**
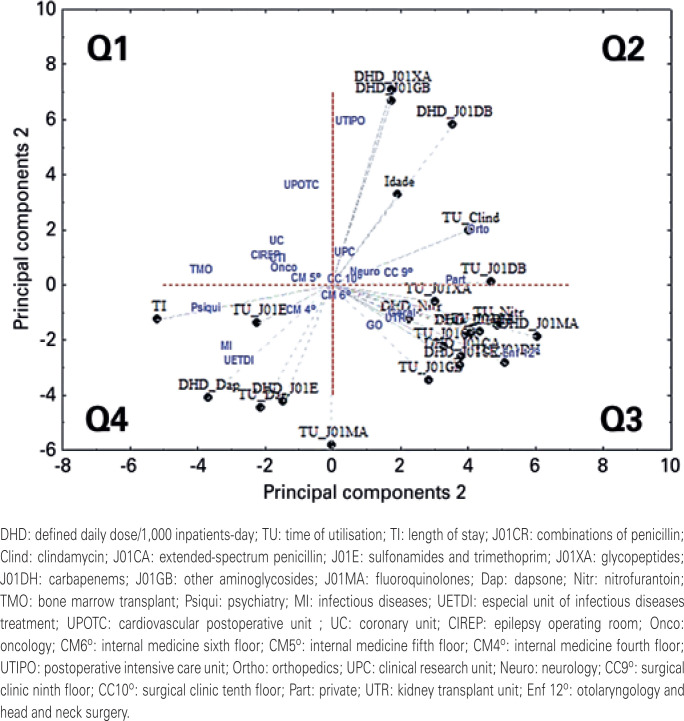
Biplot of the principal components according to specialty ward and group of antibiotic utilization (defined daily dose/1,000 inpatients-day). The closer the principal components in the chart, the more similar the behaviour of the data

The PCA reduces this multidimensional chart to two-dimensional (two-axis) chart. Figure 1 shows that after ACP data processing, what percentage each variable has or explains the variation of the total data. The two dimensions of the graph presented in figure 2 are the two variables that contain the greatest representation of data variation, and the points in the graphs represent the behaviour of the variable. The closer the points are on the chart, the more similar is the behaviour regarding the use of antimicrobials at the hospital. This process of mathematization and graphical representation is proven by observing the gross values.

We chose to perform this analysis due to the large number of variables, which would be unfeasible for graphical representation, interpretation and decision making. Although multivariate analysis has existed since the 1980's, and is applied in different areas, including health sciences, most studies still explore little such analysis. This proposed approach meets the current scenario that generates huge amounts of daily data for analysis, hindering interpretations and logical organization of information.^(^^13^^,^^14^^)^

The number of principal components used for interpretation considered the minimum explanation of 80% of the total variance of the data and eigenvalues above one.^(^^15^^)^

### Ethical approval

This research was approved *ad referendum* by the Research Ethics Committee (REC) of the *Faculdade de Ciências Farmacêuticas de Ribeirao Preto* , protocol 1.124.047, CAAE: 44399715.4.0000.5403, and by the CEP of *Hospital das Clínicas da Faculdade de Medicina de Ribeirão Preto* , protocol 1.139.544, CAAE: 44399715.4.3001.5440.

## RESULTS

In this study, 7,287 inpatients received 90,475 antimicrobial prescriptions. Of these inpatients, 71.1% (5,182), corresponding to 12,971 prescriptions, were prescribed prophylactic prescriptions. Among these inpatients, 55.5% (2,875) were female. The mean age of all inpatients with prophylactic prescriptions was 51.3 years (SD of 17.3).

There were 5,819 hospitalisations among these inpatients, corresponding to a ratio of 1.12 hospitalisations per inpatient. The average length of stay was 12.8 days, but 71.4% of hospitalisations had a length of stay of fewer than 7 days.

More than a half of clinical diagnosis from these inpatients is represented by three classifications from ICD-10, neoplasms (26.7%), musculoskeletal system and connective tissue (13.6%), and diseases of circulatory system (10.1%).

The antimicrobial prophylaxis utilisation represented 11.7% of total hospital consumption (treatments more prophylaxis). Cefazolin was the most commonly used, corresponding to 52.0% of prophylaxis utilisation. In addition, the orthopedic, thoracic and cardiovascular postoperative unit (UPOTC), and postoperative intensive care unit (UTIPO) specialty wards comprised 56.3% of all prophylaxis antimicrobial prescriptions ( Table 1 ).

**Table 1:** Specialty ward utilization of each antimicrobial class according to defined daily dose/1,000 inpatients-day

ATC class (DDD/1,000 inpatients-day)	UPOTC	UTIPO	Orthopedics	OCP	CC9^th^	CC10^th^	GO	Others
Extended-spectrum penicillin	0.0	0.0	5.3	53.3	0.0	1.5	0.6	4.2
Combinations of penicillin	0.0	0.0	1.0	16.7	0.5	0.4	0.6	7.8
First-generation cephalosporin	206.6	268.9	309.5	38.1	109.2	46.3	74.5	186.8
Third-generation cephalosporin	1.6	25.5	1.9	20.1	3.6	72.3	2.0	22.8
Sulfonamides and trimethoprim	0.7	1.4	2.2	1.0	0.6	4.5	0.7	78.3
Lincosamides	0.0	9.4	3.3	54.2	1.0	0.0	1.7	13.9
Other aminoglycosides	30.3	142.6	46.8	1.5	11.5	1.8	2.4	66.5
Fluoroquinolones	0.0	0.0	14.4	17.8	15.0	7.5	5.9	29.1
Glycopeptides	31.6	41.4	30.8	2.8	3.1	5.5	1.0	30.8
Others	1.2	0.0	4.7	3.4	1.9	0.9	7.4	31.8

ATC: anatomical therapeutic chemical; DDD: defined daily dose; UPOTC: thoracic and cardiovascular postoperative unit; UTIPO: postoperative intensive care unit; OCP: otolaryngology and head and neck surgery; CC9^th^: surgical clinic ninth floor; CC10^th^: surgical clinic tenth floor; GO: gynecology and obstetrics.

Of particular note was the high utilisation of antimicrobial drugs, including aminoglycosides, in the orthopedics ward. In addition, the otolaryngology and head and neck surgery had a high rate of prescriptions of many antimicrobial classes, such as extended-spectrum penicillin, lincosamide, and first-generation cephalosporin. Finally, the surgical clinic on the tenth floor had high use of third-generation cephalosporins.

Regarding the duration antimicrobial use of these specialty wards, the orthopedics ward used SAP for less than 2 days only for penicillin and extended-spectrum penicillin combinations ( Table 2 ). In addition, third-generation cephalosporins and fluoroquinolones were used for more than 2 days in all specialty wards except for the UPOTC.

**Table 2:** Length of prophylatic utilisation time for each antimicrobial class in days, as per anatomical therapeutic and chemical, according to specialty ward

ATC classes (days)	UPOTC	UTIPO	Orthopedics	OCP	CC9^th^	CC10^th^	GO
Extendedspectrum penicillins	0.0±0.0	0.0±0.0	1.8±0.9	1.8±0.7	0.0±0.0	1.2±0.5	1.4±0.5
Combinations of penicillins	0.0±0.0	0.0±0.0	1.8±1.3	1.7±0.9	2.0±1.0	1.7±1.1	2.0±0.7
First-generation cephalosporins	1.7±0.6	1.4±0.6	2.3±1.8	1.4±0.7	1.8±1.1	1.4±0.8	1.1±0.4
Third-generation cephalosporins	1.0±0.0	2.3±1.1	2.4±2.9	1.9±0.9	2.6±1.4	2.3±1.1	1.7±0.7
Sulfonamides and trimethoprim	1.0±0.0	2.0±0.0	5.0±5.9	1.7±1.5	1.6±0.5	4.9±4.0	2.8±2.2
Lincosamides	0.0±0.0	4.0±0.0	2.4±2.0	2.2±1.3	1.4±0.5	0.0±0.0	1.7±0.7
Other aminoglycosides	1.4±0.6	1.1±0.4	2.7±2.2	2.0±1.4	1.4±0.6	1.1±0.3	2.1±1.4
Fluoroquinolones	0.0±0.0	0.0±0.0	2.3±1.6	2.3±2.2	2.0±1.0	2.8±2.5	3.6±4.1
Glycopeptides	1.5±0.6	1.6±0.5	2.2±2.0	1.5±0.6	1.6±0.7	1.2±0.5	2.1±1.4

Results expressed as mean ± standard deviation of the duration (days).

ATC: anatomical therapeutic chemical; UPOTC: thoracic and cardiovascular postoperative unit; UTIPO: postoperative intensive care unit; OCP: otolaryngology and surgery of head and neck; CC9^th^: surgical clinic ninth floor; CC10^th^: surgical clinic tenth floor; GO: gynecology and obstetrics.

Regarding the exploratory analysis of principal components, five factors were the most important to explain 80% of variation in antibiotic utilization in each specialty ward: DDD/1,000 inpatients-day of combinations of penicillins, clindamycin, extended-spectrum penicillin, glycopeptides, and duration of sulfonamide and trimethoprim utilisation ( Figure 1 ).

The bone marrow transplant, psychiatry, infectious diseases, special unit of infectious disease treatment, coronary unit, epilepsy operating room, oncology, internal medicine fifth floor, and internal medicine fourth floor wards had similar length of stay, duration of dapsone and sulfonamides and trimethoprim utilisation, and DDD/1,000 inpatients day of dapsone and sulfonamides and trimethoprim utilisation ( Figure 2 , quadrants Q1 and Q4). The infectious diseases and special unit of infectious disease treatment wards were in the same quadrant ( Figure 2 , quadrant 4).

The specialty wards of UTIPO, orthopedics, clinical research unit, neurology, and surgical clinic ninth floor had similar duration of clindamycin use; first-generation cephalosporin use; patient age; and DDD/1,000 inpatient-day utilisation of glycopeptides, other aminoglycosides, and first-generation cephalosporin ( Figure 2 , quadrant Q2).

## DISCUSSION

Among 22 hospital specialty wards, three (UTIPO, UPOTC, and orthopedics) were responsible for more than half of the SAP utilisation.

Although cardiothoracic surgeries are considered clean surgeries, SAP is indicated because patients are usually vulnerable, many are diabetic, and the procedure is lengthy.^(^^16^^)^ Several clinical trials have showed no benefit associated with the prolonged use of SAP compared to one dose in coronary artery bypass graft,^(^^17^^,^^18^^)^ cardiac valve surgery,^(^^17^^)^ and in patients with severe heart failure who could not be weaned from cardiopulmonary bypass without intra-aortic balloon pumping.^(^^19^^)^

In our study, the duration of first-generation cephalosporin utilisation in the UPOTC was 1.7 day (SD of 0.6). In addition, we noted the utilisation of aminoglycosides for SAP. Clinical trials have shown that safer drugs with a low spectrum of activity, such as cefazolin, are effective for the prevention of SSI in cardiovascular surgeries, and is recommended by international guidelines, except for conditions with a high risk of multidrug bacterial resistance colonisation.^(^^17^^,^^18^^,^^20^^)^

In a Jordanian study,^(^^21^^)^ 58.9% of cardiac surgeries had a SAP duration longer than recommended by international guidelines, but 95,8% of cardiac surgeries complied with these guidelines when choosing antibiotics for surgical prophylaxis. In France,^(^^22^^)^ 48.0% of SAP duration were longer than recommended, and 92.3% of antibiotic choices were as recommended.

The intensive care unit is a specialty ward that receives patients in critical condition, and patients in this unit have a high risk of infectious disease due to reduced immunity in the patient population, in addition to the use of invasive devices, such as catheters or tubes for mechanical ventilation.^(^^23^^)^

Nevertheless, the recommendation for SAP use in the intensive care unit is the same as that for any surgery in non-critical patients. This unit performs many types of surgical procedures; thus, the antibiotic class use may vary. We noted a high use of first-generation cephalosporins, which are recommended for most types of surgical procedures; glycopeptide for patients allergic to penicillin, or in settings with high frequency of methicillin-resistant S *taphylococcus aureus* ; and aminoglycosides for intestinal surgeries.^(^^20^^)^

However, regarding the duration of utilisation, the recommendation is one dose of SAP.^(^^20^^)^ This prescription behaviour was not observed in our study, neither in a study developed in a surgical intensive care unit in Germany, which found SAP utilization for 2 to 3 weeks after cerebrospinal shunts, corresponding 1,030 DDD/1,000 inpatients-day.^(^^24^^)^

Orthopedic surgeries are considered clean; hence, there is no evidence of benefit from SAP utilisation, except in surgeries with prosthesis implantation or major surgeries, or in immunosuppressed patients or emergency surgery. In these cases, SAP is recommended. One dose of cefazolin with additional doses, according to the procedure length or bleeding volume, is recommended in orthopedic surgery because of low cost, low toxicity, and good serum and bone tissue levels of the drug.^(^^25^^)^

Among the specialty wards, orthopedics was the one that most used SAP, particularly aminoglycosides. This antimicrobial class is not indicated for this type of surgery.^(^^20^^)^ However, during the study period, there was an outbreak of SSI by *Gram* -negative bacilli in spinal surgeries, forcing the Committee on Control and Use of Antimicrobials to recommend the addition of gentamicin to cefazolin for the SAP in these procedures.

Buckley et al.,^(^^26^^)^ evaluated if the duration of SAP utilisation in hip fracture surgery influenced the incidence of SSI. They did not find a significant difference between the group that used one dose of cefazolin and three doses of placebo, and the group that used four doses of cefazolin. Queiroz et al.,^(^^6^^)^ assessed 3 months of SAP utilisation in the orthopedics ward after the implementation of a SAP protocol. They reported 105.0 DDD/1,000 inpatient-days of SAP utilisation. In addition, the only drug used was cefazolin. In Singapore, the median length of time of SAP use was 3 days, similar to that found in our study.^(^^27^^)^

Lower use was observed in the comparisons of the SAP utilisation of the ninth and tenth floor surgical clinics, otolaryngology and head and neck surgery, gynecology and obstetrics and orthopedics wards, in addition to UPOTC and UTIPO. However, this utilisation could be further reduced if the duration of SAP utilisation corresponded to that described in the literature. In these specialty wards, we observed 2 or more days of SAP for some antimicrobial classes.

Clindamycin is indicated for head and neck surgeries, thus representing the class most often used in this specialty ward. Carrol et al.,^(^^28^^)^ studied the prophylactic use of clindamycin in head and neck surgeries and observed no difference in SSI between the groups that used a single dose or 5 days of SAP. A study from Taiwan on head and neck surgeries reported similar findings.^(^^29^^)^ Nonetheless, in practice, we observed postoperative use as studied in United Kingdom, 70% of surgeons used 3 days or more of SAP in laryngectomy.^(^^30^^)^

Regarding gynecological and obstetric surgeries, the scientific literature reports the use of many types of drugs for SAP with similar effectiveness: clindamycin combined with aztreonam or cefotaxime,^(^^31^^)^ cefoperazone combined with sulbactam,^(^^32^^)^ cefazoline^(^^33^^)^ and ampicillin more metronidazole.^(^^34^^)^ Despite this variety of drugs, all studies showed that a single dose is as effective as the administration of SAP for multiple days. However, we did not observe this finding in our study and around the world, as we found in study from India, the mean duration of SAP utilisation was 6.14 days in gynecological and obstetric surgeries.^(^^7^^)^

In some patients with neoplasms and with human immunodeficiency virus, the prophylactic use of antimicrobial compounds could have occurred in patients not undergoing surgery. This was shown in the principal component analysis, in which we observed a similar profile of prophylactic antimicrobial use of sulfonamides and trimethoprim and dapsone in the bone marrow transplant, oncology, infectious diseases, and special unit of infectious disease treatment wards. These drugs are indicated for primary and secondary prophylaxis of pneumocystis or toxoplasmosis in immunosuppressed patients. This pathological characteristic is common in these specialty wards.^(^^35^^)^

The descriptive nature of this study prevented analysis of the causes that motivated the misuse of SAP and its consequences. In addition, the use of the secondary database prevented the complete control of information by the researchers, resulting in an information bias.

Yet, descriptive studies are the best strategy to determine the profile of SAP utilisation; therefore, we can propose a hypothesis for testing in future analytical studies. In addition, descriptive studies allow the assessment of all study population with a low cost and time but with significative conclusions.^(^^36^^)^

Few descriptive studies have assessed the general SAP utilisation in the specialty hospital wards in Brazil. Therefore, this study provided a comprehensive overview on the current scenario of antimicrobial utilisation abuse, and the emergence of antimicrobial resistance that concern public health worldwide.

## CONCLUSION

Despite the study limitations, it is clear that surgical antibiotic prophylaxis utilisation is inappropriate and does not consider World Health Organization guidelines or hospital protocols. The presence of the Antimicrobial Use Committee and Hospital Infection Control Committee in this hospital was not sufficient to avoid this scenario. Therefore, additional strategies are required to promote and evaluate the rational use of surgical antibiotic prophylaxis, including audit teams, frequent training on surgical antibiotic prophylaxis protocols, and more radical measures, such as restriction of surgical antibiotic prophylaxis prescriptions that do not follow the protocol, mainly at orthopedics, postoperative intensive care unit, orthopedic, thoracic and cardiovascular postoperative unit, gynecology and obstetrics, and otolaryngology units.
