# Supplementary material for: Surgical antibiotic prophylaxis: is the clinical practice based on evidence?
Source: Einstein (Sao Paulo). 2020 Nov 12;18:eAO5427. doi: 10.31744/einstein_journal/2020AO5427 (PMC7664826; doi:10.31744/einstein_journal/2020AO5427)
Supplement: Translation

# Translation: Antibioticoprofilaxia cirúrgica: sua prática clínica está baseada em evidências?

**Authors:** Lucas Borges Pereira, Cinara Silva Feliciano, Diego Silva Siqueira, Fernando Bellissimo-Rodrigues, Leonardo Régis Leira Pereira

**Affiliations:** 1 Ribeirão PretoSP Brasil Faculdade de Ciências Farmacêuticas de Ribeirão Preto, Universidade de São Paulo, Ribeirão Preto, SP, Brasil.; 2 Ribeirão PretoSP Brasil Hospital das Clínicas, Faculdade de Medicina de Ribeirão Preto, Universidade de São Paulo, Ribeirão Preto, SP, Brasil.; 3 AraraquaraSP Brasil Faculdade de Ciências Farmacêuticas, Universidade Estadual Paulista “Júlio de Mesquita Filho”, Araraquara, SP, Brasil.; 4 Ribeirão PretoSP Brasil Faculdade de Medicina de Ribeirão Preto, Universidade de São Paulo, Ribeirão Preto, SP, Brasil.

**Keywords:** Revisão de uso de medicamentos, Antibioticoprofilaxia, Resistência microbiana a medicamentos, Infecção da ferida cirúrgica/prevenção & controle, Controle de infecções

## Abstract

**Objetivo:**

Avaliar a utilização de antibioticoprofilaxia cirúrgica.

**Métodos:**

Foi realizado um estudo descritivo em um hospital universitário de cuidado terciário por meio de coleta de dados de prescrição, sociodemográficos e de hospitalização sobre todos os pacientes internados em 2014 que utilizaram pelo menos um medicamento antimicrobiano. Esses dados foram coletados da base de dados eletrônica do hospital. O consumo de antimicrobianos foi analisado de acordo com a classificação anatômica terapêutica e química/dose diária definida por mil pacientes-dia. Realizou-se uma análise exploratória por meio da análise de componentes principais.

**Resultados:**

Um total de 5.182 pacientes internados receberam prescrição de antibioticoprofilaxia cirúrgica, que corresponde a 11,7% do total de antibióticos utilizados no hospital. As unidades de ortopedia, pós-operatória de cirurgia torácica e cardiovascular e terapia intensiva pós-operatória foram responsáveis pela utilização de mais da metade (56,3%) da antibioticoprofilaxia cirúrgica. A duração de uso desses antimicrobianos nessas unidades foi 2,2, 2,0 e 2,4 dias, respectivamente. Cefalosporinas de terceira geração e fluoroquinolonas foram as classes de antimicrobianos com tempo de utilização mais longo.

**Conclusão:**

A utilização de antibioticoprofilaxia cirúrgica foi inadequada nas unidades de ortopedia, pós-operatória de cirurgia torácica e cardiovascular, terapia intensiva pós-operatória, ginecologia e obstetrícia e otorrinolarigonlogia. Portanto, são importantes o desenvolvimento e a implantação de estratégias que promovam o uso racional de antibioticoprofilaxia cirúrgica nos hospitais.

## INTRODUÇÃO

A infecção do sítio cirúrgico (ISC) é facilmente evitada, caso os critérios de prevenção sejam rigorosamente seguidos, mas continua sendo uma infecção relacionada à assistência à saúde (IRAS) frequente, sendo dispendiosa para o sistema público de saúde.^(^^1^^)^

Um método de prevenção da ISC é a antibioticoprofilaxia cirúrgica (APC), que demonstrou ser efetiva na literatura científica.^(^^2^^,^^3^^)^No entanto, o benefício é obtido apenas quando os seguintes critérios são utilizados corretamente: administração pré-cirúrgica; seleção adequada do antimicrobiano com base no tipo de procedimento cirúrgico e no patógeno mais frequentemente observado na ISC; administração de mais de uma dose em procedimentos de maior duração; e descontinuação da dose após o fechamento da ferida operatória.^(^^1^^,^^4^^)^

Apesar disso, a APC tem sido utilizada por longos períodos, ou são prescritos antimicrobianos de amplo espectro, o que não oferece benefício adicional para determinadas cirurgias ou para procedimentos sem tal indicação. Zhang et al.,^(^^5^^)^observaram que foi administrada APC 2 horas antes da incisão cirúrgica, com tempo de utilização variando de 1 a 14 dias. Queiroz et al.,^(^^6^^)^relataram que apenas 3,3% das prescrições de APC estavam isentas de erros. Mesmo com os protocolos hospitalares de APC, existem discrepâncias na utilização de antimicrobianos, conforme relatado por Khakhkhar et al.,^(^^7^^)^e Schmitt et al.^(^^8^^)^

A eficácia da APC é determinada pelas concentrações plasmáticas de agentes antimicrobianos que impedem o crescimento microbiano durante os procedimentos cirúrgicos. A utilização prolongada após a cirurgia não oferece benefício adicional na prevenção da ISC e está associada à resistência antimicrobiana.^(^^1^^,^^9^^)^

A resistência antimicrobiana tem sido discutida por órgãos governamentais e de saúde pública. A 71ª Assembleia Geral das Nações Unidas abordou a disseminação de infecções resistentes a antimicrobianos, e a Organização Mundial da Saúde (OMS) divulgou o documento *Antimicrobial Resistance: Global Report on Surveillance* , destacando a importância da utilização racional de antimicrobianos para evitar resistência.^(^^1^^,^^10^^)^

Os poucos relatórios sobre a utilização de APC, além desse contexto de resistência antimicrobiana, enfatizam a necessidade de estudos sobre a utilização de medicamentos como estratégia para seu uso racional, a fim de melhorar a efetividade da profilaxia cirúrgica e reduzir a seleção de resistência antimicrobiana.

## OBJETIVO

Descrever a utilização da antibioticoprofilaxia cirúrgica e avaliar se ela está de acordo com as diretrizes internacionais.

## MÉTODOS

### Sujeitos e cenário

Este estudo descritivo foi realizado em um hospital universitário de nível terciário de atenção à saúde com 706 leitos, no estado de São Paulo. Os dados do paciente eram gerenciados por sistemas eletrônicos, que incluíam a Prescrição Eletrônica e o Sistema de Informações Hospitalares. A população estudada compreendeu todos os pacientes internados com idade ≥18 anos que receberam prescrição antimicrobiana entre 1° de janeiro e 31 de dezembro de 2014.

### Coleta de dados

Os sistemas eletrônicos do hospital foram analisados, sendo coletados os dados de todos os antimicrobianos prescritos em 2014. Neste estudo, foram analisados apenas antimicrobianos prescritos com objetivo de profilaxia, utilizados para prevenir a infecção de pacientes internados, como a ISC em pacientes cirúrgicos (essas informações foram apresentadas em dados do sistema eletrônico). As informações coletadas sobre a prescrição incluíram a dose, o tempo de utilização, a enfermaria onde o medicamento foi prescrito, o nome e a identificação hospitalar do paciente. Os dados clínicos incluíram o tempo de internação, o diagnóstico clínico de acordo com a Classificação Estatística Internacional de Doenças e Problemas Relacionados à Saúde (CID-10) e o número de pacientes internados por dia, em 2014, para cada enfermaria.

### Análise estatística

Foi realizada análise descritiva dos dados dos pacientes internados. Os resultados das análises descritivas foram apresentados como frequências absolutas e relativas, média e desvio padrão (DP).

A utilização de antimicrobianos foi calculada por meio da dose diária definida (DDD) por mil pacientes-dia. Esse cálculo foi realizado para cada medicamento utilizado em cada enfermaria.^(^^11^^)^

$$\text{DDD}/1.000\text{pacientes}-\text{dia}=\frac{\text{Soma de antimicrobianos usados em 2014}\left(\text{em gramas}\right)}{\text{DDD do antimicrobiano}\times \text{pacientes}-\text{dia da enfermaria em 2014}}\times 1,000$$

Os antimicrobianos foram classificados de acordo com a classificação anatômica terapêutica química (ATC) padronizada pela OMS.^(^^12^^)^

Para complementar a avaliação da utilização de antimicrobianos, uma análise exploratória dos dados foi realizada pela análise de componentes principais (ACP) usando o programa Statistica^®^7.0. As variáveis incluídas nessa análise foram tempo de utilização do medicamento, DDD/1.000 pacientes-dia, idade do paciente e tempo de internação. A ACP é uma técnica estatística multivariada exploratória. Ela utiliza um processo de análise matricial para converter um conjunto de dados com muitas variáveis em um novo conjunto de dados representado por vetores. Esses novos vetores representam a interação entre as diferentes variáveis, reduzindo a quantidade de dados a serem analisados, sem perder a representatividade do banco de dados original. Após a padronização, os dados foram codificados como zero para a média e 1 para a variância. O objetivo é reduzir a dimensionalidade dos dados, para que eles sejam representados geometricamente.

O banco de dados do estudo tem as variáveis e o consumo de antimicrobianos das enfermarias de cada classe ATC. Se representássemos esse consumo antimicrobiano de cada enfermaria em um gráfico, precisaríamos de um gráfico multidimensional (múltiplos eixos), pois cada classe ATC é uma dimensão.

A ACP reduz esse gráfico multidimensional, de modo que se torne um gráfico bidimensional (dois eixos). A figura 1 mostra, após o processamento dos dados pela ACP, qual percentagem cada variável possui ou explica a variação dos dados totais. As duas dimensões do gráfico apresentadas na figura 2 são as duas variáveis com maior representatividade na variação dos dados, e os pontos nos gráficos representam o comportamento da variável. Quanto mais próximos no gráfico estiverem os pontos, mais semelhante é o comportamento em relação ao uso de antimicrobianos no hospital. Esse processo de matematização e representação gráfica é comprovado pela observação dos valores brutos.

**Figura 1:** Variância das enfermarias (A) explicada pelos componentes principais, representados pelo consumo de cada grupo de antibióticos (B)

**Figura 2:** Biplotagem dos componentes principais de acordo com a enfermaria e o grupo de utilização de antibióticos (dose diária definida/1.000 pacientes-dia). Quanto mais próximos os componentes principais estiverem no gráfico, mais semelhante é o comportamento dos dados

Optamos por realizar essa análise devido ao grande número de variáveis, o que seria inviável para representação gráfica, interpretação e tomada de decisão. Embora a análise multivariada exista desde a década de 1980 e seja aplicada em diferentes áreas, inclusive nas ciências da saúde, a maioria dos estudos ainda explora pouco essas análises. Essa abordagem proposta atende ao cenário atual, que gera enormes quantidades de dados diários para análise, dificultando interpretações e uma organização lógica das informações.^(^^13^^,^^14^^)^

O número de componentes principais utilizados para interpretação considerou a explicação mínima de 80% da variância total dos dados e autovalores acima de um.^(^^15^^)^

### Aprovação ética

Esta pesquisa foi aprovada *ad referendum* pelo Comitê de Ética em Pesquisa (CEP) da Faculdade de Ciências Farmacêuticas de Ribeirão Preto, parecer 1.124.047, CAAE: 44399715.4.0000.5403, e pelo CEP do Hospital das Clínicas da Faculdade de Medicina de Ribeirão Preto, parecer 1.139.544, CAAE: 44399715.4.3001.5440.

## RESULTADOS

Neste estudo, 7.287 pacientes internados receberam 90.475 prescrições de antibióticos. Desses pacientes, 71,1% (5.182), correspondendo a 12.971 prescrições, receberam prescrições profiláticas. Dentre esses pacientes, 55,5% (2.875) eram do sexo feminino. A média da idade de todos os pacientes com prescrições profiláticas foi de 51,3 anos (DP de 17,3).

Ocorreram 5.819 internações entre esses pacientes, correspondendo a uma proporção de 1,12 hospitalização por paciente. O tempo médio de permanência foi de 12,8 dias, mas 71,4% das internações tiveram tempo de permanência inferior a 7 dias.

Mais da metade dos diagnósticos clínicos desses pacientes internados foi representada por três classificações da CID-10: neoplasias (26,7%), sistema músculo-esquelético e tecido conjuntivo (13,6%) e doenças do sistema circulatório (10,1%).

A utilização de profilaxia antimicrobiana representou 11,7% do consumo total hospitalar (tratamento mais profilaxia). A cefazolina foi a mais usada, correspondendo a 52,0% da utilização profilática. Além disso, a enfermaria de ortopedia, a unidade pós-operatória de cirurgia torácica e cardiovascular (UPOTC) e a unidade de terapia intensiva pós-operatória (UTIPO) corresponderam a 56,3% de todas as prescrições antimicrobianas profiláticas ( Tabela 1 ).

**Tabela 1:** Utilização de cada classe de antimicrobianos nas enfermarias, de acordo com dose diária definida/1.000 pacientes-dia

Classe ATC (DDD/1.000 pacientes-dia)	UPOTC	UTIPO	Ortopedia	OCP	CC 9°	CC 10°	GO	Outros
Penicilinas de amplo espectro	0,0	0,0	5,3	53,3	0,0	1,5	0,6	4,2
Combinação de penicilinas mais inibidor de betalactamase	0,0	0,0	1,0	16,7	0,5	0,4	0,6	7,8
Cefalosporinas de primeira geração	206,6	268,9	309,5	38,1	109,2	46,3	74,5	186,8
Cefalosporinas de terceira geração	1,6	25,5	1,9	20,1	3,6	72,3	2,0	22,8
Sulfonamidas e trimetoprim	0,7	1,4	2,2	1,0	0,6	4,5	0,7	78,3
Lincosamidas	0,0	9,4	3,3	54,2	1,0	0,0	1,7	13,9
Outros aminoglicosídeos	30,3	142,6	46,8	1,5	11,5	1,8	2,4	66,5
Fluoroquinolonas	0,0	0,0	14,4	17,8	15,0	7,5	5,9	29,1
Glicopeptídeos	31,6	41,4	30,8	2,8	3,1	5,5	1,0	30,8
Outros	1,2	0,0	4,7	3,4	1,9	0,9	7,4	31,8

ATC: classificação anatômica terapêutica química; DDD: dose diária definida; UPOTC: unidade pós-operatória de cirurgia torácica e cardiovascular; UTIPO: unidade de terapia intensiva pós-operatória; OCP: otorrinolaringologia e cirurgia de cabeça e pescoço; CC9°: clínica cirúrgica do nono andar; CC10°: clínica cirúrgica do décimo andar; GO: ginecologia e obstetrícia.

Destaca-se a elevada utilização de medicamentos antimicrobianos, inclusive aminoglicosídeos, na enfermaria de ortopedia. Além disso, a otorrinolaringologia e a cirurgia de cabeça e pescoço tiveram elevada taxa de prescrição de muitas classes de antibióticos, como penicilina de amplo espectro, lincosamida e cefalosporina de primeira geração. Por fim, a clínica cirúrgica do décimo andar apresentou alto uso de cefalosporinas de terceira geração.

Em relação ao tempo de utilização de antimicrobianos nessas enfermarias, a enfermaria de ortopedia utilizou APC por menos de 2 dias apenas para combinações de penicilina mais inibidor de betalactamase e penicilina de amplo espectro ( Tabela 2 ). Além disso, as cefalosporinas de terceira geração e as fluoroquinolonas foram usadas por mais de 2 dias em todas as enfermarias, exceto na UPOTC.

**Tabela 2:** Tempo de utilização profilática de cada classe de antimicrobianos em dias, conforme classificação anatômica terapêutica e química, de acordo com a enfermaria

Classes ATC (dias)	UPOTC	UTIPO	Ortopedia	OCP	CC 9°	CC 10°	GO
Penicilinas de amplo espectro	0,0±0,0	0,0±0,0	1,8±0,9	1,8±0,7	0,0±0,0	1,2±0,5	1,4±0,5
Combinação de penicilinas mais inibidor de betalactamase	0,0±0,0	0,0±0,0	1,8±1,3	1,7±0,9	2,0±1,0	1,7±1,1	2,0±0,7
Cefalosporinas de primeira geração	1,7±0,6	1,4±0,6	2,3±1,8	1,4±0,7	1,8±1,1	1,4±0,8	1,1±0,4
Cefalosporinas de terceira geração	1,0±0,0	2,3±1,1	2,4±2,9	1,9±0,9	2,6±1,4	2,3±1,1	1,7±0,7
Sulfonamidas e trimetoprim	1,0±0,0	2,0±0,0	5,0±5,9	1,7±1,5	1,6±0,5	4,9±4,0	2,8±2,2
Lincosamidas	0,0±0,0	4,0±0,0	2,4±2,0	2,2±1,3	1,4±0,5	0,0±0,0	1,7±0,7
Outros aminoglicosídeos	1,4±0,6	1,1±0,4	2,7±2,2	2,0±1,4	1,4±0,6	1,1±0,3	2,1±1,4
Fluoroquinolonas	0,0±0,0	0,0±0,0	2,3±1,6	2,3±2,2	2,0±1,0	2,8±2,5	3,6±4,1
Glicopeptídeos	1,5±0,6	1,6±0,5	2,2±2,0	1,5±0,6	1,6±0,7	1,2±0,5	2,1±1,4

Resultados expressos como média ± desvio padrão do tempo (dias).

ATC: classificação anatômica terapêutica química; UPOTC: unidade pós-operatória de cirurgia torácica e cardiovascular; UTIPO: unidade de terapia intensiva pós-operatória; OCP: otorrinolaringologia e cirurgia de cabeça e pescoço; CC9°: clínica cirúrgica do nono andar; CC10°: clínica cirúrgica do décimo andar; GO: ginecologia e obstetrícia.

Em relação à análise exploratória dos componentes principais, cinco fatores foram os mais importantes para explicar 80% da variação na utilização de antibióticos em cada enfermaria: DDD/1.000 pacientes-dia de combinações de penicilinas mais inibidor de betalactamase, clindamicina, penicilina de amplo espectro, glicopeptídeos e tempo de utilização de sulfonamida e trimetoprim ( Figura 1 ).

As enfermarias de transplante de medula óssea, psiquiatria, doenças infecciosas, unidade especial de tratamento de doenças infecciosas, unidade coronariana, centro de cirurgia de epilepsia, oncologia, clínica geral do quinto andar e clínica geral do quarto andar apresentaram tempo de permanência, tempo de utilização da dapsona e de sulfonamidas e trimetoprim e DDD/1.000 pacientes-dia de dapsona e sulfonamidas e trimetoprim semelhantes ( Figura 2 , quadrantes Q1 e Q4). As enfermarias de moléstias infecciosas e a unidade especial de tratamento de doenças infecciosas estavam no mesmo quadrante ( Figura 2 , quadrante 4).

As enfermarias UTIPO, ortopedia, unidade de pesquisa clínica, neurologia e clínica cirúrgica do nono andar tiveram tempo de uso de clindamicina, uso de cefalosporina de primeira geração, idade do paciente e DDD/1.000 pacientes-dia de glicopeptídeos, outros aminoglicosídeos e cefalosporina de primeira geração semelhantes ( Figura 2 , quadrante Q2).

## DISCUSSÃO

Dentre as 22 enfermarias do hospital, três (UTIPO, UPOTC e ortopedia) foram responsáveis por mais da metade das utilizações da APC.

Embora as cirurgias cardiotorácicas sejam consideradas cirurgias limpas, a APC é indicada porque o paciente internado geralmente está vulnerável, muitos têm diabetes e o procedimento é demorado.^(^^16^^)^Vários ensaios clínicos não mostraram benefício associado ao uso prolongado da APC em comparação à dose única na cirurgia de revascularização do miocárdio,^(^^17^^,^^18^^)^na cirurgia valvar cardíaca^(^^17^^)^e em pacientes com insuficiência cardíaca grave que não puderam ser desmamados da circulação extracorpórea sem bombeamento com balão intra-aórtico.^(^^19^^)^

Em nosso estudo, o tempo de utilização de cefalosporina de primeira geração na UPOTC foi de 1,7 dia (DP de 0,6). Além disso, notamos a utilização de aminoglicosídeos na APC. Os ensaios clínicos mostraram que medicamentos mais seguros com baixo espectro de atividade, como a cefazolina, são efetivas para a prevenção da ISC em cirurgias cardiovasculares, sendo recomendados pelas diretrizes internacionais, exceto em situações com alto risco de colonização por bactérias com resistência a múltiplos fármacos.^(^^17^^,^^18^^,^^20^^)^

Em estudo realizado na Jordânia,^(^^21^^)^58,9% das cirurgias cardíacas tiveram tempo de utilização da APC mais longo do que o recomendado pelas diretrizes internacionais, mas 95,8% das cirurgias cardíacas seguiram essas diretrizes na escolha de antibióticos para a profilaxia cirúrgica. Na França,^(^^22^^)^48,0% das cirurgias tiveram tempo de utilização da APC maior que o recomendado, e 92,3% das opções de antibióticos foram as recomendadas.

A unidade de terapia intensiva é uma unidade que recebe pacientes em estado crítico, com elevado risco de apresentar doença infecciosa devido à imunidade reduzida, além do uso de dispositivos invasivos, como cateteres ou tubos para ventilação mecânica.^(^^23^^)^

No entanto, a recomendação para uso da APC na unidade de terapia intensiva é a mesma que para qualquer cirurgia realizada em pacientes que não estejam em estado crítico. Essa unidade realiza muitos tipos de procedimentos cirúrgicos, assim, a classe de antibióticos utilizada pode variar. Observamos elevada utilização de cefalosporinas de primeira geração, as quais são recomendadas para a maioria dos tipos de procedimentos cirúrgicos; de glicopeptídeos para pacientes alérgicos à penicilina ou em locais com frequência elevada de *Staphylococcus aureus* resistente à meticilina e de aminoglicosídeos para cirurgias intestinais.^(^^20^^)^

Contudo, em relação ao tempo de utilização, a recomendação é de uma dose de APC.^(^^20^^)^Essa conduta de prescrição não foi observada em nosso estudo, nem em estudo desenvolvido em unidade de terapia intensiva cirúrgica da Alemanha, que constatou a utilização de APC por 2 a 3 semanas após cirurgia de derivação cefalorraquidiana, correspondendo a 1.030 DDD/1.000 pacientes-dia.^(^^24^^)^

As cirurgias ortopédicas são consideradas limpas, e não há evidências de benefício para a utilização da APC, exceto em cirurgias com implante de próteses, em grandes cirurgias, em pacientes imunodeprimidos ou em cirurgias de emergência. Nesses casos, a APC é recomendada. Uma dose de cefazolina com doses adicionais, de acordo com a duração do procedimento ou o volume de sangramento, é recomendada em cirurgia ortopédica, devido ao baixo custo, à baixa toxicidade e aos bons níveis séricos e em tecido ósseo do medicamento.^(^^25^^)^

Dentre as enfermarias, a ortopedia foi a que mais utilizou APC, principalmente aminoglicosídeos. Essa classe antimicrobiana não é indicada para esse tipo de cirurgia.^(^^20^^)^No entanto, durante o período do estudo, houve um surto de ISC por bacilos *Gram* -negativos na coluna vertebral, forçando a Comissão de Controle e Uso de Antimicrobianos a recomendar a adição de gentamicina à cefazolina para a APC nesses procedimentos.

Buckley et al.,^(^^26^^)^avaliaram se o tempo de utilização da APC na cirurgia de fratura de quadril influenciou na incidência de ISC. Não encontraram diferença significativa entre o grupo que usou dose de cefazolina e três doses de placebo e o grupo que usou quatro doses de cefazolina. Queiroz et al.,^(^^6^^)^avaliaram 3 meses de utilização da APC na enfermaria de ortopedia após a implementação de um protocolo de APC. Relataram a utilização de 105,0 DDD/1.000 pacientes-dia de APC. Além disso, o único medicamento utilizado foi a cefazolina. Em Cingapura, a média do tempo de uso de APC foi de 3 dias, semelhante ao encontrado em nosso estudo.^(^^27^^)^

Ao serem comparadas com a ortopedia, a UPOTC e a UTIPO, observa-se reduzido consumo de APC nas clínicas cirúrgicas do nono e décimo andares, otorrinolaringologia e cirurgias de cabeça e pescoço e na ginecologia e obstetrícia. No entanto, essa utilização pode ser reduzida ainda mais se o tempo de utilização da APC correspondesse ao descrito na literatura. Nessas enfermarias, observamos 2 ou mais dias de APC para algumas classes de antimicrobianos.

A clindamicina é indicada para cirurgias de cabeça e pescoço, representando a classe mais usada nessa enfermaria. Carrol et al.,^(^^28^^)^estudaram o uso profilático da clindamicina em cirurgias de cabeça e pescoço e não observaram diferença na ISC entre os grupos que usaram uma dose única ou 5 dias de APC. Estudo realizado em Taiwan sobre cirurgias de cabeça e pescoço relatou achados semelhantes.^(^^29^^)^Porém, na prática, observamos o uso pós-operatório como estudado no Reino Unido, onde 70% dos cirurgiões usaram por 3 dias ou mais a APC na laringectomia.^(^^30^^)^

Em relação às cirurgias ginecológicas e obstétricas, a literatura científica relata o uso de muitos tipos de medicamentos para APC com efetividade semelhante: clindamicina combinada com aztreonam ou cefotaxima,^(^^31^^)^cefoperazona combinada com sulbactam,^(^^32^^)^cefazolina^(^^33^^)^e ampicilina mais metronidazol.^(^^34^^)^Apesar dessa variedade de medicamentos, todos os estudos mostraram que uma dose única é tão efetiva quanto a administração de APC por vários dias. Contudo, não observamos esse achado em nosso estudo e em todo o mundo, como em estudo realizado na Índia, o tempo médio de utilização da APC foi 6,14 dias em cirurgias ginecológicas e obstétricas.^(^^7^^)^

Em alguns pacientes com neoplasias e vírus da imunodeficiência humana, uso profilático de antimicrobianos pode ter ocorrido em pacientes não submetidos à cirurgia. Isso foi demonstrado na ACP, na qual observamos perfil semelhante do uso antimicrobiano profilático de sulfonamidas, trimetoprim e dapsona nas enfermarias de transplante de medula óssea, oncologia, doenças infecciosas e na unidade especial de tratamento de doenças infecciosas. Esses medicamentos são indicados para profilaxia primária e secundária de pneumocistose ou toxoplasmose em pacientes imunodeprimidos. Essa característica patológica é comum nessas enfermarias.^(^^35^^)^

A natureza descritiva deste estudo impediu a análise das causas que motivaram o uso indevido de APC e suas consequências. Além disso, o uso de fonte secundária de banco de dados impediu o controle completo das informações pelos pesquisadores, resultando em um viés de informação.

Estudos descritivos, porém, são a melhor estratégia para determinar o perfil de utilização de APC. Assim, podemos propor uma hipótese para teste em futuros estudos analíticos. Além disso, estudos descritivos permitem avaliar toda a população estudada com baixo custo e tempo, porém com conclusões significativas.^(^^36^^)^

Poucos estudos descritivos avaliaram a utilização geral da APC nas enfermarias dos hospitais do Brasil. Portanto, este estudo forneceu uma visão abrangente do cenário atual de abuso de utilização de antimicrobianos e do surgimento de resistência antimicrobiana que preocupa a saúde pública em todo o mundo.

## CONCLUSÃO

Apesar das limitações do estudo, fica claro que a utilização da antibioticoprofilaxia cirúrgica é inadequada e não leva em consideração as diretrizes ou os protocolos hospitalares da Organização Mundial da Saúde. A presença das Comissões de Uso de Antimicrobianos e Controle de Infecção Hospitalar neste hospital não foi suficiente para evitar esse cenário. Portanto, são necessárias estratégias adicionais para promover e avaliar o uso racional da antibioticoprofilaxia cirúrgica, incluindo equipes de auditoria, treinamento frequente em relação a utilização dos protocolos de antibioticoprofilaxia cirúrgica e medidas mais radicais, como a restrição de prescrições de antibioticoprofilaxia cirúrgica que não sigam o protocolo, principalmente nas unidades de ortopedia, na unidade de terapia intensiva pós-operatória, na unidade pós-operatória de cirurgia torácica e cardiovascular, nas unidades de ginecologia e obstetrícia e nas unidades de otorrinolaringologia.
